# *ADDME – Avoiding Drug Development Mistakes Early*: central nervous system drug discovery perspective

**DOI:** 10.1186/1471-2377-9-S1-S1

**Published:** 2009-06-12

**Authors:** Katya Tsaioun, Michel Bottlaender, Aloise Mabondzo, the Alzheimer's Drug Discovery Foundation

**Affiliations:** 1 Apredica, 313 Pleasant Street, Watertown, MA 02472, USA; 2 CEA, I2BM, Service Hospitalier Frédéric Joliot, Orsay, 91401, France; 3 CEA, iBiTec-S, Service de Pharmacologie et d'ImmunoAnalyse, Gif-sur-Yvette, 91191, France

## Abstract

The advent of early absorption, distribution, metabolism, excretion, and toxicity (ADMET) screening has increased the attrition rate of weak drug candidates early in the drug-discovery process, and decreased the proportion of compounds failing in clinical trials for ADMET reasons. This paper reviews the history of ADMET screening and its place in pharmaceutical development, and central nervous system drug discovery in particular. Assays that have been developed in response to specific needs and improvements in technology that result in higher throughput and greater accuracy of prediction of human mechanisms of absorption and toxicity are discussed. The paper concludes with the authors' forecast of new models that will better predict human efficacy and toxicity.

## Introduction

The goal of this paper, which was presented at the Alzheimer's Drug Discovery Foundation conference in Washington, DC on 3 February 2009 [1] is to briefly review advances in the science of predicting human absorption, distribution, metabolism, excretion and toxicity (ADMET) from *in vitro *and *in vivo *models, to review presently available assays as well as those in the process of being validated, and to present a model for designing effective *in vitro *ADMET programs that are used to advance drug-development programs at the lead-optimization and preclinical candidate selection stages.

Drug attrition late in clinical development or involving those already on the market is a serious economic problem in the pharmaceutical industry [2]. The cost of getting a drug to market is approaching US$1 billion and the cost of advancing a compound to phase I trials can reach up to US$100 million according to the Tufts Center for the Study of Drug Development, Tufts University School of Medicine [3]. The study also estimates that each day a drug is in the development stage costs US$37,000 in direct out-of-pocket costs and represents opportunity costs of US$1.1 million in lost revenue [3]. Given these huge expenditures, substantial savings can accrue from early recognition of problems that would make a compound unlikely to succeed in development [4].

The costs associated with withdrawing a drug from the market are even higher. For example, consider the case of terfenadine, which is a potent hERG ligand and is metabolized by Cyp3A4. Terfenadine was frequently co-administered with ketoconazole or erythromycin [5], both of which are Cyp3A4 inhibitors. The consequent overload resulted in increases in plasma terfenadine to levels that caused cardiac toxicity [6]. This toxicity caused terfenadine to be withdrawn from the market [7] at an estimated cost of US$6 billion. Another example is the broad-spectrum antibiotic trovafloxacin, which was introduced in 1997 and soon became Pfizer's top seller. It is metabolically activated *in vivo *and forms a highly reactive metabolite, which resulted in severe drug-induced hepatotoxicity [8]. Trovafloxacin was black labeled in 1998 [9], potentially costing Pfizer US$8.5 billion in lawsuits [10]. With the ADMET assays now available, the liabilities associated with these drugs could have been recognized in early preclinical development.

The purpose of preclinical ADMET is to eliminate weak candidates. This allows drug-development resources to be focused on fewer but more-likely-to-succeed drug candidates. In 1993, 40% of drugs failed in clinical trials because of pharmacokinetics (PK) and bioavailability problems [11]. Since then, major technological advances have occurred in molecular biology and screening to allow major aspects of ADMET to be assessed much earlier, at the lead-optimization stage. By the late 1990s the pharmaceutical industry as a whole recognized the value of early ADMET assessment and began routinely employing it. The results were striking. ADME and drug metabolism PK reasons for failure fell from 40% to 11% [4]. Now, lack of efficacy and human toxicity are the major reasons for failure [12].

The terms 'drugability' and 'druglikeness' were coined by Dr Christopher Lipinski, who also proposed what has come to be known as Lipinski's 'Rule of 5' due to the frequent appearance of '5' in the rules [13]. The Rule of 5 has come to be a compass for the drug discovery industry [14]. It stipulates that small-molecule drug candidates must have: a molecular weight less than 500 g/mol; a partition coefficient (logP – a measure of hydrophobicity) less than 5; no more than 5 hydrogen bond donors; no more than 10 hydrogen bond acceptors. A compound with fewer than three of these properties is unlikely to become an orally bioavailable drug. It is worth noting here that there are exceptions to Lipinski's Rule of 5 that have become marketed drugs, such as drugs that are taken up by active transport mechanisms, natural compounds, oligonucleotides and proteins.

The drug-discovery industry is experiencing dramatic structural change. It is no longer just the domain of traditional large pharmaceutical companies. Now venture-capital-funded startups, governments, venture philanthropy and other nonprofit and academic organizations are significant participants in the search for new drug targets, pathways, and molecules. Thus, it is becoming increasingly important to ensure that investors, donors, and taxpayers' money is efficiently used so that new drugs for unmet medical needs may be delivered to the public. ADMET profiling has been proven to weed out poor drug candidates and to speed discovery and development.

While lack of efficacy and unexpected toxicity are the major causes of drug failure in clinical trials, as discussed above, a prime determinant of both is how the drug penetrates biological barriers such as the cell membrane, intestinal wall, or blood-brain barrier (BBB). This is especially true in central nervous system (CNS) drugs, because candidates that have *in vitro *efficacy but cannot penetrate the BBB to reach targets in the brain are unlikely to have *in vivo *efficacy in patients. Thus, the delivery of systemically administered drugs to the CNS of mammals is limited by the presence of the BBB, as the BBB effectively isolates the brain from the blood because of the presence of tight junctions connecting the endothelial cells of the brain vessels. In addition, specific metabolizing enzymes and efflux pumps, such as P-glycoprotein (P-gp) and the multi-drug-resistance protein (MRP), located within the endothelial cells, actively remove exogenous molecules from the brain [15,16].

Hence, it is no coincidence that CNS drugs under development have a notoriously high failure rate [17]. In recent years, 9% of compounds that entered phase I survived to launch, and only 3% to 5% of CNS drugs made it to market [17]. Over 50% of this attrition resulted from failure to demonstrate efficacy in phase II studies. Over the past decade, phase II failures have increased by 15%. Compounds with demonstrated efficacy against the target *in vitro *and in animal models have more often than not proved ineffective in human disease. Many of these failures may occur due to drugs not reaching the CNS target because of lack of BBB permeability.

Thus, given the extraordinary cost of new drug development, it would be highly desirable to have effective, cost-efficient high-throughput tools to measure BBB permeability before proceeding to expensive compound- and time-consuming animal BBB permeability studies or, worse yet, failing in clinical trials. Correspondingly, with such *in vitro *tools, promising drug candidates without effective BBB penetration could be improved at the earliest stages of development to increase intrinsic permeability by, for example, removing structural components that mediate interaction(s) with efflux proteins, and/or lowering binding to brain tissue [18].

The development of drugs targeting the CNS requires precise knowledge of the drug's brain penetration. Ideally, this information would be obtained as early as possible to focus resources on compounds most likely to reach the target organ. The physical transport and metabolic composition of the BBB is highly complex. Numerous *in vitro *models have been designed to study kinetic parameters in the CNS, including non-cerebral peripheral endothelial cell lines, immortalized rat brain endothelial cells, primary cultured bovine, porcine or rat brain capillary endothelial cells and co-cultures of primary brain capillary cells with astrocytes [19-21]. *In vitro *BBB models must be carefully assessed for their capacity to reflect accurately the passage of drugs into the CNS *in vivo*.

Alternatively, several *in vivo *experimental setups have been used to estimate BBB passage of drugs directly in laboratory animals. *In vivo *transport across the BBB was first studied in the 1960s using the early indicator diffusion method of Crone [22]. Other *in vivo *techniques were later proposed: brain uptake index measurement [23], the *in situ *brain perfusion method [24,25], and autoradiography and intracerebral microdialysis [26] are in current use as well. In spite of the sophisticated equipment, technical expertise and mathematical modeling that these models require, all of them have important limitations, notably species differences and low throughput, as well as their invasiveness. These limitations render them unsuitable for use during early stages of drug discovery and development.

Hence, *in vitro *and *in vivo *models alike remain mere approximations of the complex human BBB, and their relevance to the real life situation must be carefully considered. The most relevant way to conduct such controlled experiments is to cross-compare the BBB passage of a series of compounds evaluated with both *in vitro *and *in vivo *models alongside each other. This enables cross-correlations of PK data and the assessment of the predictive power of *in vitro *and *in vivo *tests.

## The evolving science of ADMET

Regulatory authorities have relied upon *in vivo *testing to predict the behavior of new molecules in the human body since the 1950s. Bioavailability, tissue distribution, PK, metabolism, and toxicity are assessed in one rodent and one non-rodent species before a drug may be tested in human safety clinical trials (phase I).

Biodistribution is assessed using radioactively labeled compounds later in development because it is expensive, both in terms of synthesizing sufficient amounts of radioactively labeled compound and performing the animal experiments [23].

Pharmacodynamic (PD) effectiveness of test compounds is normally first assessed through *in vitro *models such as receptor binding, followed by confirmation through *in vivo *efficacy models in mice or rats. The predictive ability of these models depends on the therapeutic area and the model itself. Infectious disease models are considered to have the best predictive ability, whereas CNS and oncology animal models are generally the least predictive of human efficacy. *In vivo *PK studies in a variety of animal models are routinely used for lead optimization to assess drug metabolism and absorption. Understanding the PK-PD relationship is crucial in developing an understanding of the mechanism of action and metabolic fate of a molecule, which help to explain and support efficacy results. However, there are significant differences in absorption and metabolism among species. Animal studies alone, therefore, may cause over- or under-prediction of absorption or metabolic degradation of new chemical entities (NCEs).

Toxicity and safety studies are performed in models that are relevant to an NCE's mode of action and therapeutic area. *In vivo *toxicity models are required for an Investigational New Drug (IND) application to the US Food and Drug Administration (FDA), but these have substantial predictive weaknesses. In a retrospective study of 150 compounds from 12 large pharmaceutical companies, the combined animal toxicity study of rodents and nonrodents accurately predicted only 50% of the human hepatotoxicity. Worse, at this poor level of accuracy, large numbers of compounds that would perhaps have been safe in human patients, but show toxicity in animals, would be removed from development without going forward into human trials [27]. The toxicity of the other approximately 50% of compounds whose toxicity could not be predicted was attributed to 'idiosyncratic human hepatotoxicity, that cannot be detected by conventional animal toxicity studies.' It has long been widely known that mechanisms for toxicity are frequently quite different between species; yet, animal testing remains the 'gold standard' for historical reasons. The FDA and other regulatory agencies are in the process of evaluating alternatives to animal testing, with the aim of developing models that are truly predictive of human mechanisms of toxicity and limiting *in vivo *toxicology testing.

## The ADMET feedback loop

As discussed above, ADMET studies have historically focused on *in vivo *assays. These are, however, time- and resource-intensive, and are generally low throughput, which caused them to be put off until later in the development process, when more resources are released to study the few molecules that have advanced to this advanced stage. With the advent of *in vitro *high-throughput screening, molecular biology and miniaturization technologies in the 1990s, early ADMET assays were developed to predict *in vivo *animal and human results, at a level of cost-effectiveness appropriate for the discovery stage. This produced a major advance in the science of ADMET, and has created a new norm that drug-discovery programs follow in advancing compounds from hit to lead, from lead to advanced lead, and on to nominated clinical candidates. Now, early in the discovery phase, using human enzymes and human-origin cells, drug-discovery programs are able to obtain highly actionable information about the drug-likeness of their new molecules, their potential to reach target organs, and early indications of known human mechanisms of toxicities. ADMET assessment of varying complexity is now routinely done on compounds that have shown *in vitro *efficacy and at the same time with or just prior to demonstrating early proof of principle *in vivo*.

The application of early ADMET is unique to each drug-discovery program. The road from discovery to IND is not a straight line. It depends on the therapeutic area, route of administration, chemical series, and other parameters. Correspondingly, the importance of the various ADMET assays depends on the specifics of the drug-discovery program. ADMET assays can also be divided into those that are routine and those reserved for more advanced profiling, with the division being a function of cost effectiveness and the need for the specific information. For instance, one does not normally need to know during the hit-to-lead phase which transporters in the gastrointestinal tract or the brain are involved in transporting the drug; however, later in the development process this issue becomes more relevant.

In some cases the FDA has moved to require some of the new *in vitro *ADMET assays. For example, *in vitro *drug-drug interaction studies may now be conducted under the guidance from the FDA dated September 2006. The guidance document defines precisely how to conduct cytochrome P450 (CYP450) inhibition and induction and P-gp interaction studies [28].

How should a discovery team employ early ADMET? The answer is not simple and formulaic: it is a process. It is useful to start from the ultimate goal and work backwards towards discovery. The drug discovery and development team should first define the target product profile, which includes indication, intended patient population, route of administration, acceptable toxicities, and ultimately will define the drug label. Of course, the target product profile invariably will evolve during the life of the project, but having its major parameters established at the start helps the team to keep their eyes on the ball and work in close collaboration between disciplines such as biology and chemistry, discovery and development, pre-clinical and clinical groups. Once the target product profile is identified, then major design elements of the phase II and III clinical trials can be outlined, which in turn lead to questions about the product's tolerable toxicity and safety, which will then define the regulatory toxicity and safety studies in animals, which will lead the team to the discovery and preclinical-development questions to be addressed via their specific early ADMET program.

In the discovery phase, at the beginning of this exciting and risky journey, how does one put this information into action? For example, if a compound has high target receptor binding and biological activity in cells and in relevant *in vivo *animal models, how can one maximize the chances of it becoming a successful drug some day? A molecule needs to cross many barriers on the way to its target. The first barrier is seemingly simple: solubility. A solubility screen will provide information about the NCE's solubility in fluids compatible with administration to humans. The next barrier is chemical and metabolic stability. Chemical stability in buffers, simulated gastric and intestinal fluids, and metabolic stability in plasma, hepatocytes or liver microsomes of different species can be measured to predict a compound's stability in the different environments it will encounter in the human body on the way to its target.

The second step is to define some of the absorption properties of the compounds. Are they likely to be bioavailable? Measurement of permeability across Caco-2 cell monolayers is a good predictor of human oral bioavailability. For CNS drugs, assessment of BBB penetration would come at this stage and is usually a part of lead optimization campaigns. Passive BBB permeability may be assessed using BBB-parallel artificial membrane permeability assays (BBB-PAMPAs), whereas potential for active uptake or efflux may be determined using *in vivo *models or relevant *in vitro *BBB models expressing efflux and influx transporters.

Measurement of binding to plasma proteins indicates the degree of availability of the free compound in the blood circulation. This is critical as only unbound drugs are able to get to the target and exert their pharmacological effects. Metabolism and drug-drug interaction issues can be detected by screening for inhibition of CYP-450 liver enzymes. All these assays allow chemists and biologists to obtain actionable information early, allowing them to gain understanding of structure-activity and structure-property relationships.

The next step of determining whether drug-drug interactions are involved is required for advanced lead optimization. The effect of drug transporters on permeability and the effect of drugs on transporter activity can be measured in Caco-2 for the impact on intestinal absorption or using relevant *in vitro *BBB models. P-gp interactions are particularly important for CNS drugs due to high expression of these efflux transporters in the human BBB. Early knowledge about these interactions is instrumental to the medicinal chemistry strategy and helps drive lead optimization. *In vivo *toxic effects on human cells can be predicted *in vitro *by measuring cytotoxicity using mammalian cell lines or primary cells. The effect of a compound on CYP450 metabolism can be identified by determining the 50% inhibitory concentration (IC_50_) for each CYP450 enzyme. These relationships between the NCE and metabolizing enzymes need to be evaluated in the context of the human effective dose and maximum effective plasma concentrations. These human data are not normally available at early stages of discovery, but could be extrapolated from animal PK/PD results for compounds in more advanced stages of development.

It is important to understand these transporter and CYP450 relationships for several reasons. First, the compound may affect the effective plasma concentrations of other drugs taken concomitantly with the compound of interest if they are metabolized by the same CYP450 enzymes (see the discussion of the terfenadine case above). Second, if the parent drug is a CYP450 inducer, it may increase the clearance rate of concomitantly administered drugs that are metabolized by these CYP450 enzymes. This may result in a decrease in these drugs' effective plasma concentrations, thus decreasing their pharmacological effect. Third, metabolites formed via CYP450 metabolism may be responsible for undesirable side effects such as organ toxicity. Fourth, the metabolite of a compound may actually be responsible for a compound's efficacy, and not the parent compound. The metabolite may even have a better efficacy, safety, and PK profile than its parent. If so, metabolism can be exploited to produce a better drug, which will substantially change the medicinal chemistry strategy. Fifth, the identification of drug-metabolizing enzymes involved in the major metabolic pathways of a compound helps in predicting the probable drug-drug interactions in humans. This information may also make human clinical trials designed to detect drug-drug interaction unnecessary, accruing a substantial cost savings in development.

ADMET is a tool that supports overall program goals. Seldom will negative results from a single ADMET assay kill a compound or a program. Remember, the Rule of 5 requires that only three of the four conditions be met, and even then there are exceptions. Instead, as was illustrated above in the case of metabolism, the results are more likely to just change the direction of the medicinal chemistry.

**Figure 1:**
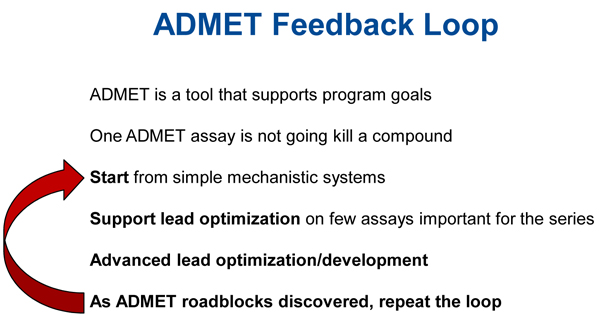
**ADMET feedback loop**.

After assessing compounds in a few simple mechanistic systems such as plasma and liver microsomal stability screens in relevant species, one moves on to lead optimization using assays to identify potential liabilities. Finally, at the stage of advanced lead optimization and development, systems that are more complex are used to more thoroughly understand a compound's metabolic fate and absorption mechanism, and this understanding is used to drive efficient development. As ADMET roadblocks are discovered, which they inevitably will be, one repeats the loop until a clear path is found (Figure 1).

### Impact of ADMET

Early ADMET not only provides the data necessary for selecting preclinical candidates, by providing crucial information to medicinal chemists, it can also accelerate the timelines for IND and subsequently new drug application (NDA) submission, which means more time on the market under patent protection and hence greater profits. For investors, this is a major parameter. For philanthropic organizations and from the standpoint of public policy, it means increasing the time of clinical benefit to the public. Data compiled by the Tufts Center for Drug Discovery have identified that, for a typical, moderately successful proprietary drug (US$350 million annual sales), each day's delay equates to US$1.1 million in lost patent-protected revenues – revenues that provide the return on investment needed to fund drug discovery [3]. Further, the shorter the discovery and development timelines are, the faster venture capital and angel investors can get to a liquidity event. As drug discovery takes longer to commercialize than any other form of product development, its slowness to produce returns is a major impediment for obtaining investment. Speeding up drug discovery and development should attract more investment in drug discovery research.

ADMET technologies remain a work in progress. There are many challenges in accurately measuring BBB penetration, which may be one of the reasons for poor human efficacy of CNS drug candidates. Another challenge is detection of all mechanisms of human idiosyncratic toxicity. These mechanisms cause the most expensive, harmful, and disheartening form of drug attrition – post-commercialization toxicity. Progress is being made. Many idiosyncratic drug reactions are due to formation of short-lived reactive metabolites that bind covalently to cell proteins [29], and the extent to which a compound will generate these can now be detected before a compound reaches human patients. Other mechanisms of human toxicity can now also be detected early in discovery. Some of the assays that are available to detect them will be briefly described in the following section.

## New ADMET tools

Penetrating the BBB is a challenge particular to CNS drug discovery. Its importance is such that it needs to be addressed first in this review. Another challenge caused by BBB permeability is that many drugs not intended as CNS therapeutics cause neurotoxicity, which should be avoided. While artificial membrane permeability assays (PAMPA and BBB-PAMPA) offer a cost-effective and high-throughput way of screening for passively absorbed compounds, they do not predict active transport in or out of the brain.

### *In vitro *model of human adult blood-brain barrier

Many new drugs designed to work in the CNS may show exceptional therapeutic promise due to their high potency at the target site, but lack general efficacy when administered systemically. In many cases, the problem is due to lack of penetration of the BBB, and this has become a major problem that has impeded the discovery and development of active CNS drugs. CEA Technologies previously reported the development of a new co-culture-based model of human BBB able to predict passive and active transport of molecules into the CNS [19]. This new model consists of primary cultures of human brain capillary endothelial cells co-cultured with primary human glial cells [19,30]. The advantages of this system are: it is made of human primary culture cells; it avoids species, age and inter-individual differences since the two cell types are removed from the same person; and it has been shown to express functional efflux transporters such as P-gp, MRP-1, MRP-4, MRP-5 and breast cancer resistance protein (BCRP).

This model shows good promise for assessment of permeability of drugs and specific transport mechanisms, which are not possible in PAMPA or other cell models due to incomplete expression of active transporters.

**Figure 2:**
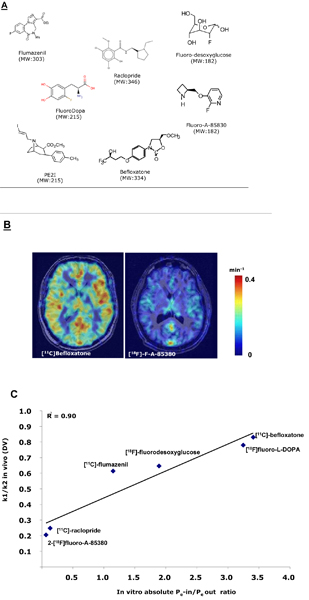
***In vitro*-*in vivo *drug transport correlation**. (A) Chemical structures of radioligands investigated and used clinically. (B) Typical imaging data. Co-registered positron emission tomography (PET)-magnetic resonance imaging (MRI) images representing the k1 (the kinetic rate constant of the free ligand from the plasma to the free ligand compartment in the brain), obtained in human after intravenous injection of [^11^C]-befloxatone (left) and [^18^F]-F-A-85380 (right). The PET images representing the k1 were acquired as follows. The PET image obtained at 1 minute post-injection (mean value between 30 and 90 s) is considered independent of receptor binding. This image (in Bq/mL) is corrected from the vascular fraction (Fv in Bq/mL, considered as 4% of the total blood concentration at 1 minute) and divided by the arterial plasma input function (AUC0-1 minute (area under curve) of the plasma concentration, in Bq × Minutes/mL). The resulting parametric image, expressed in min^-1^, represents an index of the k1 parameter of the radiotracer. (C)*In vivo *distribution volume (DV) as a function of the *in vitro *P_e_-in (permeability from apical to basolateral compartment)/P_e_-out (permeability from basal to apical compartment) ratio (Q). The regression line was calculated, and correlation was estimated by the two-tailed non-parametric Spearman test.

One important step in the development of any *in vitro *model is to cross-correlate *in vitro *and *in vivo *data in order to validate experimental models and to assess the predictive power of the techniques [31]. This human BBB model has been validated against a 'gold standard' *in vivo *model and has shown an excellent *in vitro-in vivo *correlation [30,32]. In this carefully designed *in vivo-in vitro *correlation study the authors reported the evaluation of the BBB permeabilities of a series of compounds studied correlatively *in vitro *using a human BBB model and *in vivo *with quantitative positron emission tomography (PET) imaging in human subjects [19]. Six clinical PET tracers with different molecular size ranges (Figure 2) and degree of BBB penetration were used (two of them [^18^F]-FDOPA and [^18^F]-FDG are ligands of amino acid and glucose transporters, respectively). The findings demonstrate that the *in vitro *co-culture model of human BBB has important features of the BBB *in vivo *(low paracellular permeability, well developed tight junctions, functional expression of important known efflux transporters) and is suitable for discriminating between CNS and non-CNS compounds. To further demonstrate the relevance of the *in vitro *human system, drug permeation into the human brain was evaluated using PET imaging in parallel with the assessment of drug permeability across the *in vitro *model of the human BBB. In this view, *in vivo *plasma-brain exchange parameters used for comparison were determined previously in humans by PET using a kinetic analysis of radiotracer binding. 2-[^18^F]fluoro-A-85380 and [^11^C]-raclopride show absent or low cerebral uptake with a distribution volume under 0.6, while [^11^C]-flumazenil, [^11^C]-befloxatone, [^18^F]-FDOPA and [^18^F]-FDG show a cerebral uptake with a distribution volume above 0.6. The *in vitro *human BBB model discriminates the compounds in the same way as *in v*ivo human brain PET imaging analysis. These data cast new light on the close relationship between *in vitro *and *in vivo *PK data (r^2 ^= 0.90, *P *< 0.001; Figure 2). Past *in vivo-in vitro *studies often did not have good correlations for substances transported into or out of the brain via active transport. This is likely because the experiments had been performed with models that either did not have adequate expression of active human transporters (such as PAMPA or MDCK cells) or had too high concentrations of compounds *in vitro*, which are known to saturate the transporters. By using radioactive labeled probes and small amounts of compounds, this problem can be avoided.

In conclusion, this *in vitro *human BBB model offers great promise of not only being developed into a reproducible screen for passive BBB permeability, but also determining active transport mechanisms. The model, due to its high-throughput potential, may help in testing large numbers of compounds of pharmaceutical importance for CNS diseases. Validation work is under way for this model (for example, [32]), where activities of transporters that are important in the BBB were assessed in a functional assay and compared between Caco-2 and human BBB models.

### Mechanisms of human toxicity

Idiosyncratic hepatotoxicity, or drug-induced liver injury, occurs in only 1 out of about 10,000 patients. Thus, it is usually statistically impossible to discover during clinical trials. Yet, in spite of its name, which literally means 'rare event with undefined mechanism', some mechanisms have now been defined. One of these is mitochondrial toxicity; the other is formation of reactive metabolites. Another mechanism of human toxicity that is not limited to the liver, but may also affect lung, spleen, and heart tissues, is phospholipidosis.

#### Mitochondrial toxicity

Mitochondrial toxicity is increasingly being implicated in drug-induced idiosyncratic toxicity. Many of the drugs that have been withdrawn from the market due to organ toxicity have been found to be mitochondrial toxicants [33]. Mitochondrial toxicants injure mitochondria by inhibiting respiratory complexes of the electron chain; inhibiting or uncoupling oxidative phosphorylation; inducing mitochondrial oxidative stress, or inhibiting DNA replication, transcription or translation [34].

**Figure 3:**
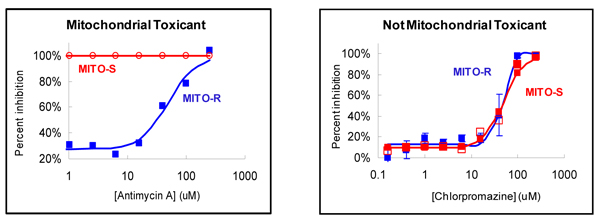
**Effect of antimycin A, a compound known to be toxic to mitochondria, and chlorpromazine on parent HepG2 cells (MITO-R, blue) and a HepG2 cell line that has been developed to become sensitive to mitochondrial toxicants (MITO-S, red)**. Left-hand side, antimycin A; right-hand side, chlorpromazine.

Toxicity testing of drug candidates is usually performed in immortalized cell lines that have been adapted for rapid growth in a reduced-oxygen atmosphere. Their metabolism is often anaerobic, by means of glycolysis, despite their having functional mitochondria and an adequate oxygen supply. On the other hand, normal cells generate ATP for energy consumption aerobically by mitochondrial oxidative phosphorylation. The anaerobic metabolism of transformed cell lines makes them less sensitive to mitochondrial toxicants, which is why mitochondrial toxicity is systematically underreported in toxicity testing in these cell lines [34,35]. To address this, HepG2 and NIH/3T3 cells can be grown in media in which glucose is replaced by galactose [33]. This changes their metabolism such that the respiratory substrate becomes more like that of normal cells, so they become more sensitive to mitochondrial toxicants without reducing sensitivity to non-mitochondrial toxicants (Figure 3).

#### Reactive metabolite formation

**Figure 4:**
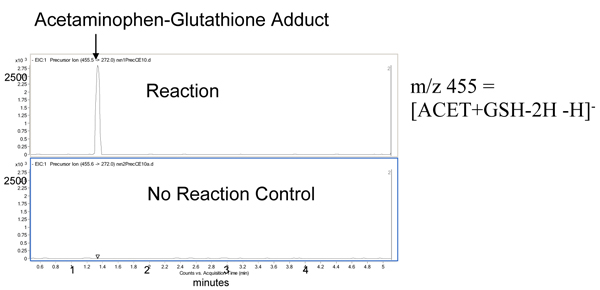
**Formation of reactive metabolites of acetaminophen**. Acetaminophen (ACET) was incubated with microsomes and glutathione (GSH) in the presence and absence of NADPH. An adduct of glutathione with acetaminophen was formed in the presence of NADPH. When NADPH was absent (no reaction control), no adduct was formed.

Another property of compounds that can cause idiosyncratic toxicity is their ability to form reactive intermediates [36]. Formation of short-lived reactive metabolites is known to be the mechanism of toxicity of some compounds, such as acetaminophen [37]. The formation of reactive metabolites can be identified by incubating test compounds with liver microsomes and adding glutathione to trap the reactive intermediates, which are then identified by liquid chromatography-tandem mass spectrometry (LC-MS/MS; Figure 4). In most programs this assay is used to assess the bioactivation potential of a compound. As with any ADMET assay, the bioactivation potential of a compound is used in the context of the whole program. Efficacy against target, clinical dose amount, dose regimen, and clinical indication will need to be considered when making decisions.

#### Phospholipidosis

**Figure 5:**
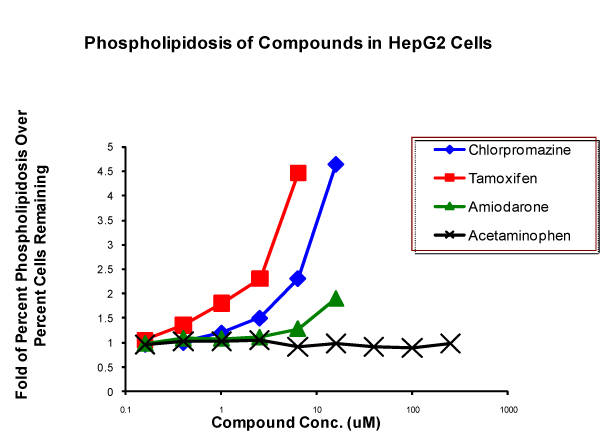
**Drug-induced phospholipidosis is determined by measuring the accumulation of a fluorescent phospholipid in cells treated with increasing drug concentrations**. Fluorescence is measured and normalized to cell number. Fluorescence is increased in cells treated with compounds that are known to cause phospholipidosis (chlorpromazine, tamoxifen, amiodarone), but it is not increased in cells treated with a compound that is known not to cause phospholipidosis (acetaminophen). Conc., concentration.

Phospholipidosis is a lysosomal storage disorder. It can be caused by drugs that are cationic amphiphiles [38]. The disorder is considered to be mild, and often resolves by itself, but drugs that cause phospholipidosis can also cause organ damage, making phospholipidosis of concern to the regulatory agencies [39]. A cell-based assay for phospholipidosis has been developed [40], which involves accumulation of a fluorescent phospholipid, resulting in an increase of fluorescence in the lysosomes of cells that have been treated with drugs that cause phospholipidosis (Figure 5). If phospholipidosis is absent, the phospholipid is degraded and fluorescence does not go up. Many of these drugs are also cytotoxic, so increases in fluorescence are normalized to cell numbers (Figure 5).

#### Genotoxicity

Genotoxicity of drugs is an important cause for concern to the regulatory authorities. The FDA recommends a number of *in vitro *and *in vivo *tests to measure the mutagenic potential of chemical compounds, including the Ames test in *Salmonella typhimurium *[41]. GreenScreen GC, a new, high-throughput assay that links the regulation of the human GADD45a gene to the production of green fluorescent protein has become available. The assay relies on the DNA damage-induced up-regulation of the *RAD54 *gene in yeast, which is measured using a promoter-green fluorescent protein fusion reporter [42]. The test is more specific and sensitive for genotoxicity than those currently recommended by the FDA, such as the Ames and mouse lymphoma tests.

## Current challenges and future directions

Great progress in the field of ADMET profiling has been made in the past 15 years. This progress has decreased the proportion of drug candidates failing in clinical trials for ADME reasons, making it a bright spot in an otherwise dismal picture of declining productivity in drug discovery. The principal barrier now is the toxicity portion of ADMET. The prediction of human-specific toxicology must be improved.

Cell-based assays using established cell lines and co-cultures have been used to determine toxicity to various organs, but many of these cell lines have lost some of the physiological activities present in normal cells. HepG2 cells, for instance, have greatly reduced levels of metabolic enzymes. Primary human hepatocytes can be used, but they are not only expensive, they suffer from high donor-to-donor variability, and they maintain their characteristics for only a short time. Three-dimensional models have been developed for cell-based therapies, including micropatterned co-cultures of human liver cells that maintain the phenotypic functions of the human liver for several weeks [43], which should provide more accurate information about toxicity when used in ADMET screening. This could be extended to other organ-specific cells, leading to development of integrated tissue models in the so-called 'human on a chip'[44]. The potential of stem cells to differentiate into cell lines of many different lineages may be exploited to develop human and animal stem-cell-derived systems for major organ systems [45].

High content screening has been used for early cytotoxicity measurement since 2003, and holds great promise. It is based on automated epifluorescence microscopy and image analysis of cells in a microtiter plate format. By using four fluorescent dyes of different colors it is possible to analyze multiple parameters at the single-cell level, including morphological and biochemical parameters that indicate pre-lethal cytotoxic effects, and represent different mechanisms of toxicity [46]. This method has been optimized for hepatocytes, and is more predictive of hepatotoxicity than currently available methods. In the future it could be applied to cells of other organs.

Molecular profiling is another alternative. It is defined as any combination or individual application of mRNA expression, proteomic, toxicogenomic, or metabolomic measurements that characterize the state of a tissue [47]. This approach has been applied in an attempt to develop profiles or signatures of certain toxicities. Molecular profiles, in conjunction with agents that specifically perturb cellular systems, have been used to identify patterns of changes in gene expression and other parameters at subtoxic drug concentrations that might be predictive of hepatotoxicity, including idiosyncratic hepatotoxicity [48]. In the future, larger data sets, high-throughput gene disruptions, and more-diverse profiling data will lead to more-detailed knowledge of disease pathways, which will facilitate making target choices and constructing detailed models of cellular systems for use in ADMET screening to identify toxic compounds early in the discovery process. The combination of *in silico*, *in vitro*, and *in vivo *methods and models into multiple content data bases, data mining, and predictive modeling algorithms, visualization tools, and high-throughput data-analysis solutions can be integrated to predict systems' ADMET properties. Such models are starting to be built and will be widely available in a decade or so [39]. The use of these tools will lead to a greater understanding of the interactions of drugs with their targets and prediction of their toxicities.

Hence, in the foreseeable future we can look forward to not only a decrease in late-stage development failures and withdrawals of marketed drugs, but also faster timelines from discovery to market, and reduced development costs through the reduction of late-stage failures.

## List of abbreviations used

ADMET: absorption, distribution, metabolism, excretion and toxicity; BBB: blood-brain barrier; CNS: central nervous system; CYP450: cytochrome P450; FDA: US Food and Drug Administration; IND: Investigational New Drug; MDCK: Madin-Darby canine kidney; MRP: multi-drug-resistance protein; NCE: new chemical entity; PAMPA: parallel artificial membrane permeability assay; PET: positron emission tomography; PD: pharmacodynamic; P-gp: P-glycoprotein; PK: pharmacokinetics.

## Competing interests

The authors declare that they have no competing interests.
